# Design and process optimization of combined medical and elderly care services: An integrated service blueprint–TRIZ model

**DOI:** 10.3389/fpubh.2022.965443

**Published:** 2022-10-13

**Authors:** An-Jin Shie, Wei-Feng Wu, Ming Yang, Xiaoji Wan, Hailin Li

**Affiliations:** ^1^College of Business Administration, Huaqiao University, Quanzhou, China; ^2^School of Economics and Management, Huaiyin Normal University, Huai'an, China; ^3^International College, Krirk University, Bangkok, Thailand; ^4^School of Management Engineering and E-Commerce, Zhejiang Gongshang University, Hangzhou, China

**Keywords:** service blueprint (SB), Fuzzy-FMEA, theory of inventive problem solving (TRIZ), combined medical and elderly care, service innovation

## Abstract

China's increasingly aging population is resulting in an imbalance between supply and demand for elderly care resources. The theory of “combined medical and elderly care” (CMEC) has introduced a new perspective in the conception of China's elderly care problems. This study employed the service blueprint, fuzzy failure mode and effects analysis (Fuzzy-FMEA), and the theory of inventive problem solving (TIPS or the Russian acronym TRIZ) for the process optimization of CMEC services in three phases. In the first phase (service process analysis), potential service failure points in the service process were analyzed using the service blueprint technique. In the second phase (service failure diagnosis), Fuzzy-FMEA was applied to diagnose the service failure modes and explore the possible causes and effects. The service failure modes were then prioritized based on fuzzy numbers and the cumulative fuzzy risk priority number (Fuzzy-RPN). Finally, in the third phase (generation of service optimization solutions), the TRIZ parameters, inventive principles, and contradiction matrix were first employed to select TRIZ inventive principles. The selected TRIZ inventive principles were then used to inspire inventive solutions for new service processes. Finally, a case study was conducted on the service processes of elderly care institutions to demonstrate the applicability of the optimization solutions.

## Introduction

According to the 14th Five-Year Plan of the Chinese government, in order to cope with the problems caused by an aging population, it is necessary to strengthen the health services for the elderly, promote “combined medical and elderly care” (CMEC) services and meet the demand for care services of elderly people with disabilities and dementia. The aging population has led to a surge in demand for elderly care services (1). According to the 2019 National Bureau of Statistics data, the number of people over 60 years old in China reached 254 million, accounting for nearly 20% of the total population (1). Furthermore, this trend of aging is expected to increase in the future. China's elderly population aged over 60 years is projected to exceed 400 million in 2035 and 500 million by 2055. Against the backdrop of an aging population and longer life expectancy, the number of elderly people living alone in China is gradually increasing, and it is insufficient to rely solely on their children for elderly care and meet their multifaceted needs (2). China's elderly care services face the risk of an imbalance between supply and demand. According to the literature, China has an average of only two beds per 100 elderly people, which is far below the elderly care standard of 5–8 beds per 100 elderly people in developed countries (3). Families of the elderly are also faced with different forms of pressure, including the provision of medical care and rehabilitation, imposing new demands on the country and society when it comes to dealing with the manifold aspects of an aging society. According to the 14th Five-Year Plan, to cope with the problems of an aging population, it is necessary to strengthen health services for the elderly, promote CMEC services, and meet the demand for elderly care services for people with disabilities and dementia, which are crucial in alleviating the associated pressures.

Owing to the lack of a robust management system in the Chinese government and the relative scarcity of professional caregivers for CMEC services in the country, problems with the design of CMEC services in relevant elderly care institutions need to be addressed. In the implementation process, there are one or more factors (e.g., service capacity, service flexibility, number of care providers, quality of care services, facilities, and equipment) that can have a decisive impact on the quality of CMEC services (2, 4, 5). Therefore, the potential of elderly care services can be fulfilled by optimizing service processes and improving and upgrading service factors. To this end, many elderly care institutions require a service design approach to analyze service failures and develop new service solutions to meet the care needs of the elderly and alleviate the imbalance and conflicting pressures on resources (4, 5).

Researchers have previously conducted studies on healthcare services similar to the concept of CMEC, such as nursing care in Japan, long-term care (LTC) in the United States, and integrated care in the United Kingdom. These studies have explored the concepts of LTC in the United States with respect to understanding the nature, determinants, social processes, and impacts of LTC (6–8); examined the rationale for integrated services in primary care settings in the UK and proposed the Rainbow Model of Integrated Care (9)^[7]^; and studied Integrated Funding Models (IFM) of sustainable integrated care for chronic conditions in Ontario, Canada (10). However, there is a lack of research on the optimization of specific service processes in CMEC.

Based on previous research, this study combined the application of service blueprint, fuzzy failure mode and effects analysis (Fuzzy-FMEA), and the theory of inventive problem solving (TRIZ) to form an integrated service blueprint-TRIZ model. The advantage of this model is that it replaces the reliance on the service designer's intuition and personal experience with a quantifiable scientific method to systematically identify failures in the service process and perform optimization. The application of this method to research on the CMEC service process may successfully fill the gaps of current research.

## Literature review

Based on the findings of previous research, this study employed the service blueprint technique to map the CMEC service delivery process to identify the CMEC points and their service failure points, aiming to improve the CMEC service process design. The possible causes and effects of failure points were then analyzed using Fuzzy-FMEA, and the Fuzzy Risk Priority Number (Fuzzy-RPN) was calculated from the fuzzy numbers obtained for prioritization. In addition, by combining TRIZ with the service blueprint technique, the causes and effects of service failures were mapped and adapted to the innovative principles and methods of TRIZ. Relevant optimization solutions were then designed to optimize the service process and support policies of CMEC. A literature review of relevant concepts is provided as follows.

### Definition of CMEC

CMEC (combined medical and elderly care) is a Chinese term, and similar concepts can be found in the healthcare services of other countries, including nursing care in Japan, LTC (long-term care) in the United States, and integrated care in the United Kingdom (4–6, 10). In the United States, Kane was the first to introduce the concept of LTC in 1987, which was defined as the provision of medical care, personal care, and social services for people with congenital or acquired disabilities (11). In 2002, the National Health Service (NHS) of the United Kingdom introduced the Integrated Care Trust to better provide integrated health services for the elderly, disabled, mentally ill, and other groups.

For LTC in China, it needs different care service deliveries, such as home care, community care, and institutional care. These care services need to be intergraded by CMEC coping with the healthy development of the aging society (12, 13). CMEC is unlike the traditional home care model in China, it focuses on integrating optimization, medical care, and pension resources for the elderly, which includes providing daily care, nursing care, medical services, and other forms from the new LTC service to achieve healthy aging (4). In China, CMEC is a new concept of elderly healthcare that combines medical service technology with elderly care services. This was proposed by professionals in various fields based on the special needs of the elderly population to improve their quality of life and adapt to the trends of an aging population. Its goal is to achieve an in-depth integration between two completely different but interrelated services, namely, medical and elderly care services (2, 4, 5). CMEC not only seeks to enhance the medical capacity of elderly care institutions, but also to relieve the pressure on elderly care resources, improve the scope and level of medical care and nursing care services for the elderly, and meet the complex needs of older adults in this era. Thus, by creating a new approach to provide elderly care through the combination of hospital care and elderly care resources, CMEC aims to achieve spatial accessibility and time continuity of medical services in the face of new demand for elderly care.

Wei and Zhang (4) explored the influence of different factors on the elderly's preferences in CMEC services by using Andersen Model for predicting the elderly's real needs, this study helps the government to better plan the elderly's pension and care services for giving full play to the important supplementary role of institutional care. Yang et al. (2) conducted a cross-sectional survey aiming at six elderly care institutions in a city in Central China according to the national guidelines for combined medical and elderly care. Their result indicated CMEC service system should increase managers' specialized training and salary for improving their elderly care skills and care service quality to deal with the diversified demands from CMEC. A long-term care education system should be integrated into CMEC by expanding the enrollment scale of the nursing school, carrying out training about elderly care skills, and issuing vocational skills certificates to those who pass the examination, the number of local nurses for the elderly will be increasing, and the quality of CMEC will be improving (14).

### Service blueprint

The service blueprint is a service analysis tool grounded in process design proposed by the American scholar Shostock in the early 1980s using a multidisciplinary approach. The service blueprint is a two-dimensional description of the service process, which divides the service process into three parts (patient actions, front-stage action, and back-stage action) to identify the line of interaction and line of visibility. This allows for a clear understanding of the interactions and influences among the different parts of the service process (15–17) while enabling the visual analysis of the root causes affecting the service perception and satisfaction of the elderly population (18).

Unlike other process maps, the service blueprint provides the service designer with a visual representation of customer service perceptions and experiences. Therefore, it is an extremely effective tool that can be used by service designers for service optimization to improve service quality, service efficiency, and customer satisfaction (15, 18). Institutionalized elderly people require long-term, continuous, diverse, and personalized services, and they can easily perceive the quality of each step in the service process. The service blueprint presents the roles of and the services provided by social workers in the institution, and identifies the areas that need improvement, which helps promote a more robust human resource system in the institution. The service designer can use the service blueprint to describe the service process to identify the parts of the service process with potential service failures. By analyzing these service failures, they can then define the possible conflicting factors in the service process and identify corresponding solutions.

### Fuzzy FMEA

FMEA is a systematic analysis tool proposed in the early 1950s. It analyzes each process of a product or service process to identify all possible failure modes and seek the causes and influencing factors of these failures. Accordingly, suitable optimization solutions are proposed through detailed analysis to prevent and control the risks of product quality or service processes and achieve the reduction of loss rate. The core tasks of FMEA involve the a priori analysis and control of product defects and service failures, as well as continuous quality improvement, elimination of possible risks and adverse effects, and improvement of the reliability of products and services during the work process (19). FMEA evaluates the severity effect of a failure mode by combining the RPN, which is the product of severity (S), occurrence probability (O), and detectability (D) (20). Among these, S refers to the degree of effect that the occurrence of a service failure mode has on the service process, O refers to the frequency at which the different failure modes occur during the service process, and D refers to the probability that the occurrence of different failure modes remains undiscovered by the relevant personnel within a fixed environment and under fixed conditions. The higher the RPN value, the higher the risk level of a given failure mode (21, 22).

However, in the process of using FMEA, there is a certain discrepancy between subjective human perception and objective facts, and this discrepancy can lead to arbitrariness, ambiguity, and uncertainty in expert judgment, which contribute to the inability of relevant theories in providing accurate data or establishing precise mathematical models for the object of study. To address this disadvantage, the previous research utilized fuzzy in FMEA. Zolfaghari and Mousavi (21) utilize the interval-valued hesitant fuzzy linguistic sets by integrating triangular fuzzy numbers to deal with group decision-making in FMEA suffering from ambiguous information. Meng et al. (23) cooperate triangular fuzzy numbers to interval-valued hesitant fuzzy linguistic sets, which can obtain both advantages of quantitative assessment in terms of interval value and quantitative assessment in terms of linguistic set. Dagsuyu et al. (24) utilize a fuzzy FMEA approach based on triangular fuzzy numbers to measure the risk priority number (RPN, composed of the severity rating, occurrence probability rating, and detection rating). The RPN is measured by five linguistic variables (inclusive of strongly disagree, disagree, neither agree nor disagree, agree, and strongly agree). These linguistic variables are suitable extraordinarily to convert into triangular fuzzy numbers (19–22).

Therefore, in this study, triangular fuzzy numbers was introduced in the FMEA method to address this deficiency. With the help of the Fuzzy-FMEA method, the uncertainty of potential service failure analysis can be resolved when applying traditional FMEA to the CMEC service process of elderly care institutions (19–22, 24). Based on the causes of failure and possible effects obtained through Fuzzy-FMEA, a series of optimization measures can be adopted to innovate and optimize the entire service process more precisely to optimize CMEC services and alleviate the pressure on elderly care resources.

### TRIZ

In 1946, Altshuller proposed the TRIZ, which was gradually shaped by summarizing the principles and innovation modes behind patented inventions. The TRIZ was initially applied in the field of engineering technology. However, with the continuous reform of the service industry, some researchers began applying TRIZ to the innovation and optimization of service processes, such as in the fields of tourism, catering, sharing economy, and e-commerce. Ilevbare et al. later proposed the TRIZ contradiction matrix, the 40 principles of invention, and other related theoretical models, tools, and research methods (25).

TRIZ is a knowledge-centered, holistic problem-solving approach that shifts the focus away from the service designer's experience and individual perceptions. It consists of many toolkits, such as problem formulation, contradiction matrix, and inventive principles, which can be applied by designers to systematically conduct the design process (15, 25). The 40 inventive principles of TRIZ and their implications have provided a new basis for understanding and applying TRIZ (26). The technical contradictions created by TRIZ can be resolved by completing the TRIZ matrix. The service designer screens for principles of resolving particular contradictions are based on the 39 engineering parameters and 40 inventive principles of TRIZ, both of which can be applied in the TRIZ method.

## Research framework

**Figure 1:**
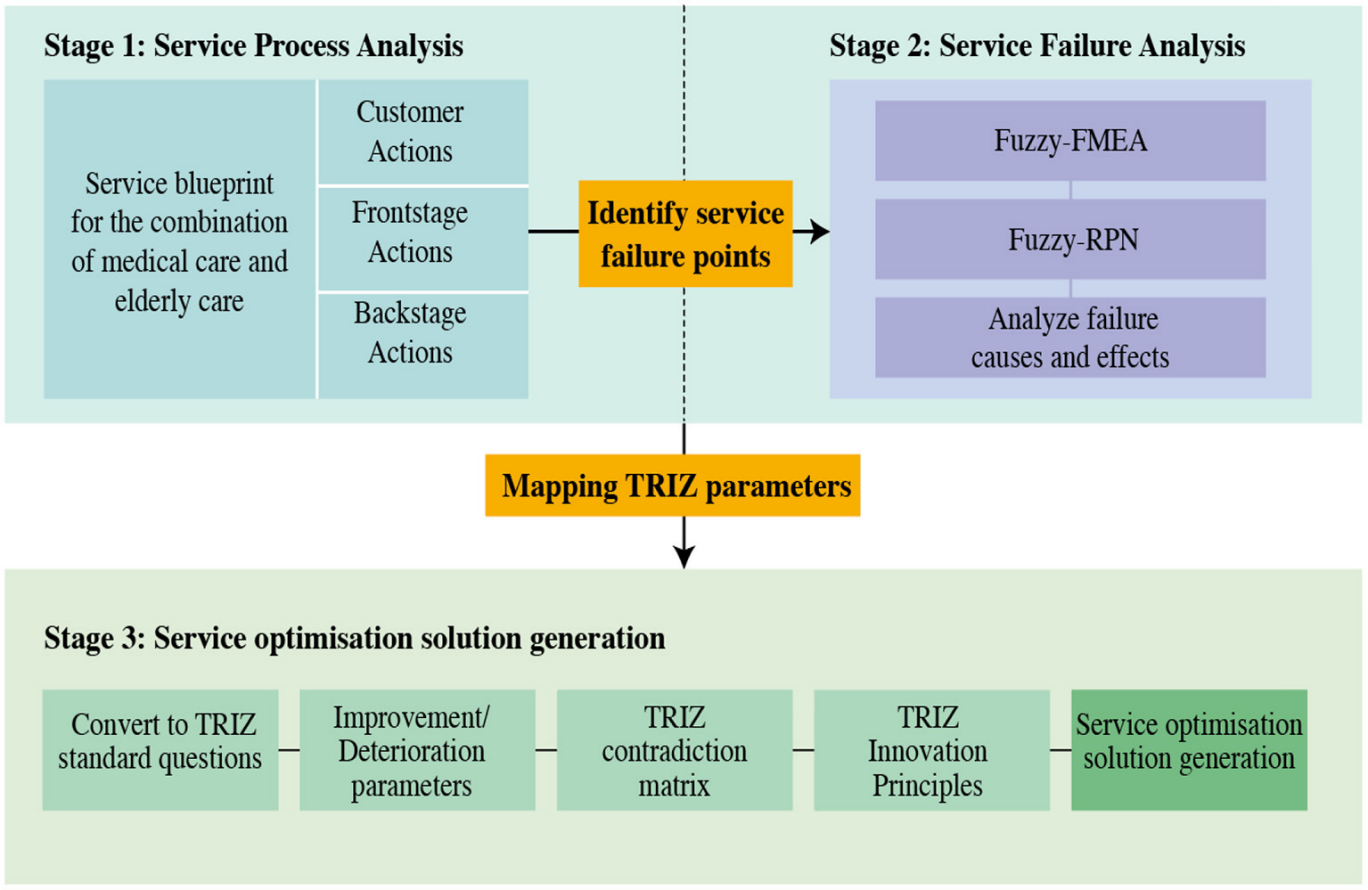
Integrated service blueprint-TRIZ model.

From the research background and literature review provided above, previous studies seem to have only employed objective and quantitative methods for analysis in the early design phase of elderly care service optimization, while neglecting systematic mechanisms for improving the service process. In fact, the CMEC service process often encounters conflicts between the special needs of the elderly and the services provided. To resolve these conflicts in the CMEC service process, a systematic approach is needed to identify potential service failures and develop solutions to improve the service process. In this study, we explored a systematic approach to service optimization design by integrating the FMEA and TRIZ models through a service blueprint (Figure 1). The research method consisted of three phases: phase I: service process analysis; phase II: service failure diagnosis; and phase III: generation of service optimization solutions.

### Phase I: Service process analysis

The purpose of this phase was to analyze the current service process for potential service failures. The service process was reviewed through field observations and interviews with service personnel. A service blueprint was then created depicting the process and indicating the service failure points of the current service process. Finally, potential service failure points were identified after the managers sought confirmation from frontline healthcare workers.

### Phase II: Service failure diagnosis

In this phase, an assessment questionnaire on potential service failures rated using a five-point scale (Appendix B) was used to gather the views of selected managerial participants. A quantitative and structural analysis was then conducted using the Fuzzy-FMEA method to diagnose potential service failures and analyze their causes and effects. To address the ambiguity and uncertainty in the opinions of the managerial participants, triangular fuzzy numbers were introduced to fuzzily the FMEA process after the collection of questionnaire data. This process is described as follows.

#### Step 1: Fuzzification of questionnaire data

To use triangular fuzzy numbers, the weights of the three factors (S, O, and D) were classified using a five-point Likert scale into five levels of semantic variables: EI (Extremely Important), VI (Very Important), I (Important), LI (A Little Important), and NI (Not Important). The membership functions of the semantic variables were normalized within {0, 1}, where the semantic variables {“EI,” “VI,” “I,” “LI,” “NI”} corresponded to the fuzzy numbers {(0.75,1,1), (0.5,0.75,1), (0.25,0.5,0.75), (0,0.25,0.5), (0,0,0.25)}, it was proposed by Bhuvanesh Kumar and Parameshwaran (27), respectively (Table 1).

**Table 1:** Correspondence table between questionnaire data and fuzzy numbers [adopted from Bhuvanesh Kumar and Parameshwaran (27)].

**Questionnaire language**			**Questionnaire score**	**Semantic variable**	**Fuzzy number**
**S**	**O**	**D**			
Catastrophic service failures	Frequent service failures	Detectable by tests after service failure occurrence of	5	EI	(0.75,1,1)
Critical service failures	Repeated service failures	Detectable once service failure has occurred	4	VI	(0.5,0.75,1)
Moderate service failures	Occasional service failures	Detectable by multiple tests before service failure occurrence	3	I	(0.25,0.5,0.75)
Minor service failures	Very few service failures	Detectable by a few tests before service failure occurrence	2	LI	(0,0.25,0.5)
Almost no service failures	Extremely unlikely service failures	Detectable without service failure occurrence or tests	1	NI	(0,0,0.25)

#### Step 2: Calculation of fuzzy number mean

After mapping the questionnaire data to fuzzy numbers, the mean values of the fuzzy numbers were calculated according to the equations given below. Let the fuzzy number S_tij_ = (q_tij_, o_tij_, p_tij_) be the triangular fuzzy number of the *j*^th^ factor of the *i*^th^ type of service failure faced by the managerial participant *t*, and *n* be the total number of managerial participants. The fuzzy number means for each factor in each type of service failure Q_ij_, O_ij_, and P_ij_ were given by equations (1), (2), and (3), respectively.


(1) $$\begin{array}{l} Q_{ij}\;=\;\frac{1}{n}\overset{n}{\underset{t\;=\;1}{\sum }}q_{tij} \end{array}$$

(2) $$\begin{array}{l} O_{ij}\;=\;\frac{1}{n}\overset{n}{\underset{t\;=\;1}{\sum }}o_{tij} \end{array}$$

(3) $$\begin{array}{l} P_{ij}\;=\;\frac{1}{n}\overset{n}{\underset{t\;=\;1}{\sum }}p_{tij} \end{array}$$

#### Step 3: Defuzzification

After calculating the fuzzy number means, The defuzzification method based on Tooranloo and Ayatollah (28) and Chen (29) 's study is applied in this study. Let X be the defuzzified value of the integrated fuzzy number for the three factors occurrence, severity, and detection (Q_i_, O_i_, P_i_). Then, the defuzzified values can be calculated with equation (4). The equation (4) was proposed by Chen (29)'s study using defuzzification method of trapezoidal fuzzy numbers. Based on the characteristic of the fuzzy number addition and multiplication operations, his formula proofed the triangular fuzzy number parametrized by the triple (e.g., Q, O, P) is equivalent to the trapezoidal fuzzy number parametrized by quadruple (e.g., Q, O, O, P). Chen (29)'s method not only considers the additivity and multiplicity in the finite data but also satisfy the unbiasedness of the finite data. Thus, the defuzzification value X of a triangular fuzzy number by (Q, O, P) is equal to


(4) $$\begin{array}{l} X\;=\;\frac{Q_{i}+O_{i}+O_{i}+P_{i}}{4} \end{array}$$

#### Step 4: Calculation of Fuzzy-RPN and prioritization

In Fuzzy-FMEA, the Fuzzy-RPN needs to be calculated to rank the risk level of service failures. After defuzzification of the questionnaire data, the Fuzzy-RPN for each service failure mode was calculated using the scores of three factors (S, O, and D), as given by equation (5).


(5) $$\begin{array}{l} \text{FRPN}\;=\;\text{S}\;\times \text{O}\times \text{D} \end{array}$$

Subsequently, prioritization of the service failure modes was performed based on the resulting Fuzzy-RPN. This phase provides a design decision-making method for potential failures, aiming to predict and detect service failures in the service process and alert managers to prioritize these failures based on their importance.

### Phase 3: Generation of service optimization solutions

In the third phase, the corresponding inventive solutions were derived from the table of TRIZ principles, which first converted the service failures in the CMEC process into standard TRIZ problems by employing the corresponding TRIZ parameters. The TRIZ contradiction matrix was then used to identify the most critical service failure models and the 40 inventive principles required as basic design elements to satisfy the service optimization. This allowed the mapping of service failures to TRIZ improving and worsening parameters. Next, the TRIZ matrix was used to select the corresponding TRIZ inventive principles. Finally, the TRIZ inventive principles obtained were screened based on an expert evaluation process, and the selected TRIZ inventive principles were then used to generate inspiration for service optimization solutions.

## Participant selection

Huai'an City, Jiangsu Province, is one of the 59 pilot cities funded by the central government for the reformation of elderly care services, and a key city in Jiangsu Province for the development of CMEC services. Therefore, Huai'an City, Jiangsu Province, was selected for this study (30, 31). By the end of 2018, more than 200 elderly care institutions were built in Huai'an City, with nearly 44,000 beds (30, 32), to cope with an aging population. These institutions provide medical services, elderly care services, and recreational facilities for the elderly. In addition, they are usually capable of providing long-term care to older adults who are unable to receive elderly care at home for various reasons.

Field surveys were first conducted in three elderly care institutions in Huai'an, followed by interviews with the managers of these institutions, to analyze the CMEC service process of elderly care institutions. Finally, the integrated service blueprint-TRIZ model was applied to the optimization of the CMEC service process in Huai'an to demonstrate the applicability of this model.

**Figure 2:**
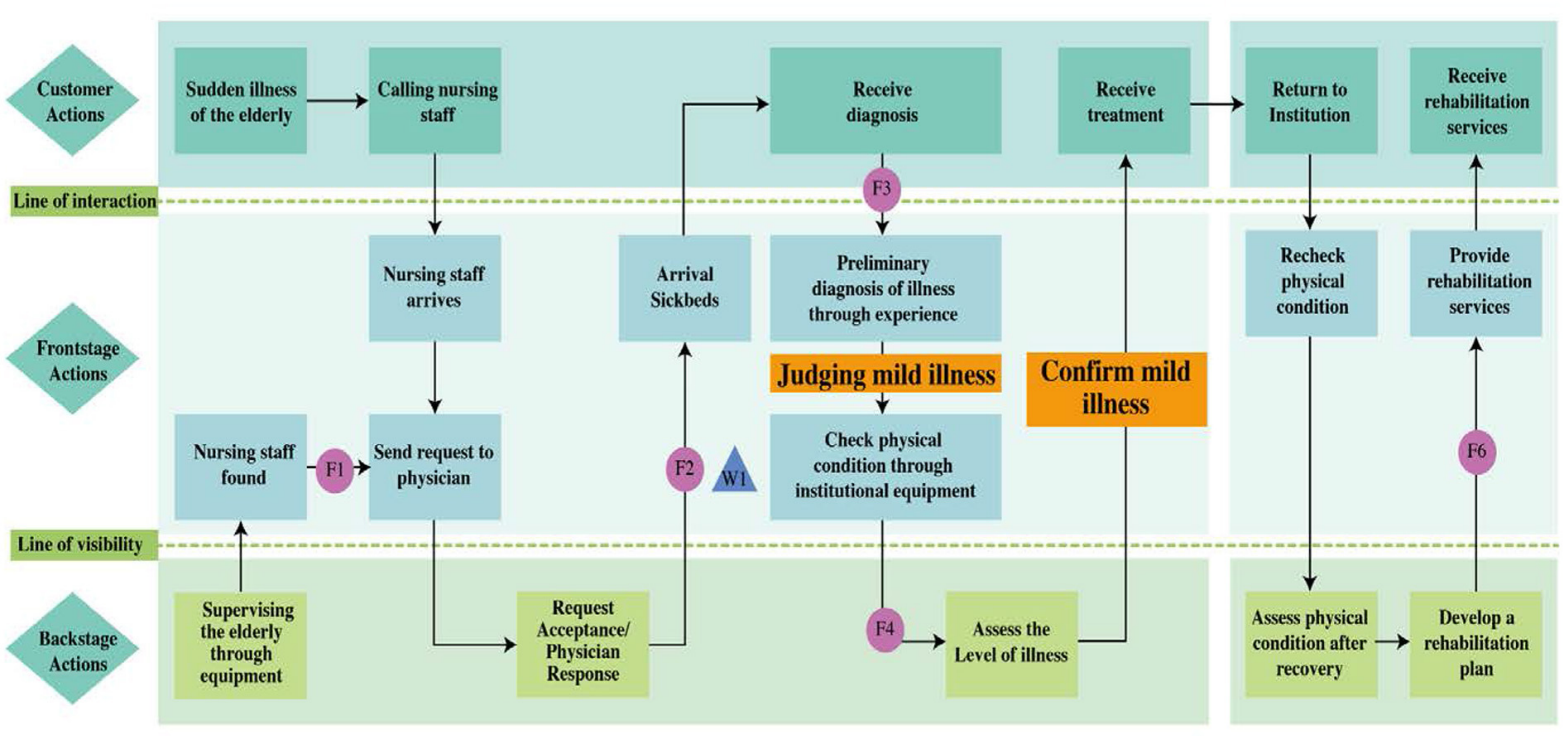
Service blueprint of CMEC for elderly people with **mild illness**.

**Figure 3:**
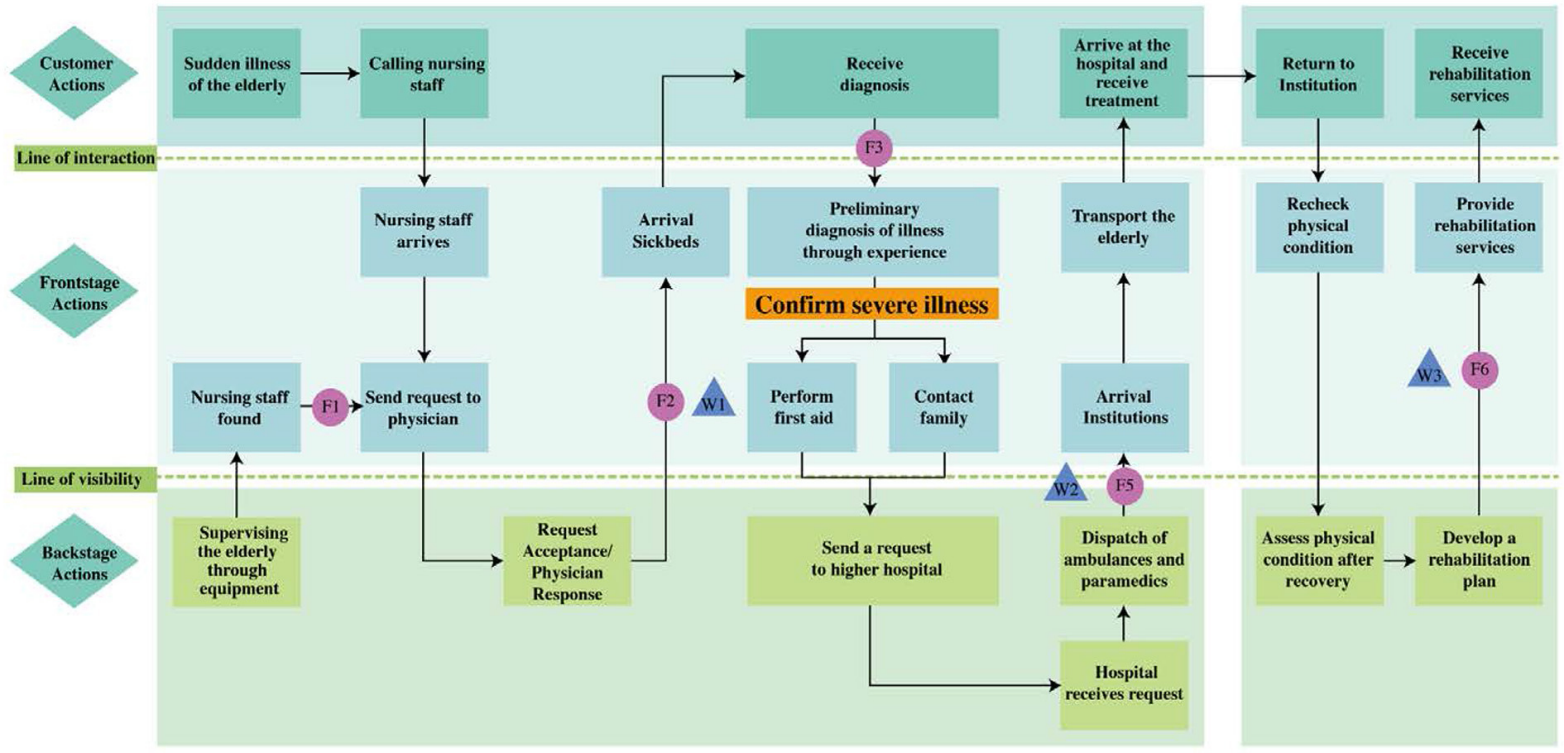
Service blueprint of CMEC for elderly people with **severe illness**.

Field surveys and interviews with front-line healthcare workers were conducted to collect the participants' real opinions and develop a service blueprint for the CMEC process (Figures 2, 3). Owing to the constraints in the medical facilities of general elderly care institutions, they can handle only a limited number of medical conditions. Therefore, patients with medical conditions beyond the capabilities of these medical facilities are transferred to hospitals. Based on the results of the interviews, this limitation was used to define the boundary between elderly patients whom the institutions were able to handle (i.e., those with mild illness) and those whom the institutions were unable to handle (i.e., those with severe illness) in the CMEC process. First, the actions of elderly patients were used to specify their behaviors and requirements. These actions included contacting nursing staff and receiving services (diagnostic services, therapeutic services, and rehabilitation services). Second, the front-stage actions of nursing staff (placed between the line of interaction and visibility) were used to specify the activities of frontline staff in providing care services to the elderly. These actions included receiving requests from the elderly, arriving at the bed, providing services, and performing rehabilitation activities. Finally, the back-stage actions of nursing staff (presented beneath the line of visibility) were used to specify the care services provided by back-stage staff in response to the elderly person's needs and to assess their health status. The key task in this phase was to identify possible service failures in the service process. After the analysis, the service failure points were presented in the service blueprint (Figures 2, 3).

In conjunction with the service blueprint shown in Table 2, six service failure points were identified: detection of sudden illness in the elderly (F1), healthcare worker's response (F2), healthcare worker's judgement of patient's condition (F3), provision of testing equipment (F4), arranging referrals (F5), and commencing rehabilitation services (F6). These potential service failures were further diagnosed in the next phase.

**Table 2:** FMEA of the CMEC model.

**No**.	**Potential service failure points**	**Service failure situation**	**Causes of failure**	**Effects of failure**
F1	Detection of sudden illness in the elderly	Human resource misallocation	Inadequate healthcare workers; Prolonged interval between ward rounds	Failure to detect the patient's illness in time, causing their condition to deteriorate
F2	Healthcare worker's response	Slow response by healthcare worker	Inadequate healthcare worker; Prolonged arrival time of healthcare workers	Inability of elderly care facilities to provide responsive services
F3	Healthcare worker's judgement of patient's condition	Error in judgement	Inexperienced healthcare workers	Failure causing the elderly to miss optimal treatment window, leading to death
^*^F4	Provision of testing equipment	Poor testing equipment	Inadequate maintenance of testing equipment; Inadequate service resources	Failure to meet the needs of the elderly; Failure causing illness in the elderly
^*^F5	Arranging referrals	Referral delays	Inadequate healthcare workers; Prolonged arrival time of ambulances	Failure to detect the patient's illness in time, leading to deterioration of their condition; Missing the optimal treatment window
F6	Commencing rehabilitation services	Inappropriate rehabilitation services	Inadequate service resources (e.g., equipment, funding and rehabilitation staff); Impatience of nursing staff	Failure leading to the degeneration and atrophy of physical functions in the elderly

* F4 is a service failure unique to minor illnesses, and ^*^F5 is a service failure unique to major illnesses.

## Empirical analysis

In the second phase, the potential service failures in the CMEC service model were analyzed using the diagnostic tool, Fuzzy-FMEA. Based on the service blueprint developed in the previous phase, individual service failure modes were identified. In the field survey, interviews were first conducted with the managers of the elderly care institutions and front-line healthcare workers, followed by the distribution of interview questionnaires covering the causes and effects of service failures to the experts, who were interviewed based on the relevant questions. The measures in the interview questionnaire included the severity rating (S), occurrence probability rating (O), and detection rating (D), which were used to analyze each service failure mode. A total of 35 interview questionnaires were administered, and these were distributed to managers and specialists conducting front-line care in each facility. The three measures in the interview questionnaires were subjective indicators evaluated by the experts based on their empirical judgments. The interview questions were highly relevant to the research questions of this study, possessing both face and content validity. The results obtained from the FMEA method did not need to follow a normal distribution, thus confirming the reliability of the data.

For each factor, a five-point Likert scale was used to measure each service failure mode. Specifically, an S rating of 1 indicated that the service failure had almost no effect, whereas 5 indicated the most severe service failure; an O rating of 1 indicated that the occurrence of the service failure was almost impossible, whereas 5 indicated that the failure was inevitable; a D rating of 1 indicated that the healthcare worker detected the service failure mode, whereas 5 indicated that the service failure could not be detected. The questionnaire data were processed using a triangular fuzzy function. The fuzzy number means were calculated using equations (1) to (3), as shown in Tables 3–5, while the defuzzified values were calculated using equation (4), as shown in Tables 6–8. The resulting Fuzzy-RPN for each service failure mode was given by equation (5) using the scores obtained from the fuzzy calculation of the participants' questionnaire ratings. Finally, the critical service failure modes were prioritized according to the magnitude of the Fuzzy-RPN, where a higher Fuzzy-RPN indicated a greater need for improvement (Table 9). Meanwhile, the comparison between traditional FMEA and Fuzzy FMEA was conducted (Table 10), and the results presented that the RPN value and prioritization in traditional and Fuzzy were similar.

**Table 3:** Fuzzy number means for S ratings in Fuzzy-FMEA.

**Category 1**		**Category 2**		**Category 3**		**Category 4**		**Category 5**		**Category 6**	
Q_1_	0.429	Q_2_	0.343	Q_3_	0.350	Q_4_	0.357	Q_5_	0.293	Q_6_	0.364
O_1_	0.679	O_2_	0.593	O_3_	0.600	O_4_	0.607	O_5_	0.543	O_6_	0.614
P_1_	0.900	P_2_	0.843	P_3_	0.843	P_4_	0.829	P_5_	0.779	P_6_	0.850

**Table 4:** Fuzzy number means for O ratings in Fuzzy-FMEA.

**Category 1**		**Category 2**		**Category 3**		**Category 4**		**Category 5**		**Category 6**	
Q_1_	0.414	Q_2_	0.400	Q_3_	0.314	Q_4_	0.357	Q_5_	0.314	Q_6_	0.364
O_1_	0.664	O_2_	0.650	O_3_	0.564	O_4_	0.607	O_5_	0.564	O_6_	0.614
P_1_	0.893	P_2_	0.871	P_3_	0.800	P_4_	0.843	P_5_	0.807	P_6_	0.843

**Table 5:** Fuzzy number means for D ratings in Fuzzy-FMEA.

**Category 1**		**Category 2**		**Category 3**		**Category 4**		**Category 5**		**Category 6**	
Q_1_	0.429	Q_2_	0.364	Q_3_	0.314	Q_4_	0.336	Q_5_	0.329	Q_6_	0.407
O_1_	0.679	O_2_	0.614	O_3_	0.564	O_4_	0.586	O_5_	0.579	O_6_	0.657
P_1_	0.893	P_2_	0.821	P_3_	0.807	P_4_	0.807	P_5_	0.807	P_6_	0.836

**Table 6:** Defuzzification of S ratings in Fuzzy-FMEA.

**NO**.	**Integrated triangular fuzzy number**			**Defuzzified value**
	**Q_**i**_**	**O_**i**_**	**P_**i**_**	
1	0.429	0.679	0.900	0.669
2	0.343	0.593	0.843	0.593
3	0.350	0.600	0.843	0.598
4	0.357	0.607	0.829	0.598
5	0.293	0.543	0.779	0.538
6	0.364	0.614	0.850	0.610

**Table 7:** Defuzzification of O ratings in Fuzzy-FMEA.

**NO**.	**Integrated triangular fuzzy number**			**Defuzzified value**
	**Q_i_**	**O_i_**	**P_i_**	
1	0.414	0.664	0.893	0.657
2	0.400	0.650	0.871	0.640
3	0.314	0.564	0.800	0.560
4	0.357	0.607	0.843	0.602
5	0.314	0.564	0.807	0.562
6	0.364	0.614	0.843	0.607

**Table 8:** Defuzzification of D ratings in Fuzzy-FMEA.

**NO**.	**Integrated triangular fuzzy number**			**Defuzzified value**
	**Q_i_**	**O_i_**	**P_i_**	
1	0.429	0.679	0.893	0.667
2	0.364	0.614	0.821	0.600
3	0.314	0.564	0.807	0.562
4	0.336	0.586	0.807	0.576
5	0.329	0.579	0.807	0.571
6	0.407	0.657	0.836	0.633

**Table 9:** Analysis of the causes of failures in the CMEC service model for the elderly.

**NO**.	**Service failure situations**	**Causes of failure**	**Defuzzified value of S rating**	**Defuzzified value of O rating**	**Defuzzified value of D rating**	**Fuzzy RPN**	**Prioritization**
F1	Human resource misallocation	Inadequate healthcare workers Prolonged interval between ward rounds	0.669	0.657	0.667	0.293	1*
F2	Slow response by healthcare workers	Inadequate healthcare workers Prolonged arrival time of healthcare workers	0.593	0.640	0.600	0.228	3*
F3	Error in judgement of patient's condition	Inexperienced healthcare workers	0.598	0.560	0.562	0.188	5
F4	Poor testing equipment	Inadequate maintenance of testing equipment Inadequate service resources	0.598	0.602	0.576	0.207	4
F5	Referral delays	Inadequate healthcare workers Prolonged arrival time of ambulances	0.538	0.562	0.571	0.173	6
F6	Inappropriate rehabilitation services	Inadequate service resources (e.g., equipment, funding and personnel) Impatience of nursing staff	0.610	0.607	0.633	0.234	2*

The symbol * indicates The top three items.

**Table 10:** Comparison of the RPN value and prioritization between traditional and Fuzzy FMEA in the CMEC service model for the elderly.

**NO**.	**Service failure situations**	**Causes of failure**	**Traditional RPN**	**Prioritization**	**Fuzzy RPN**	**Prioritization**
F1	Human resource misallocation	Inadequate healthcare workers Prolonged interval between ward rounds	44.73	1*	0.293	1*
F2	Slow response by healthcare workers	Inadequate healthcare workers Prolonged arrival time of healthcare workers	39.865	3*	0.228	3*
F3	Error in judgement of patient's condition	Inexperienced healthcare workers	34.32	6	0.188	5
F4	Poor testing equipment	Inadequate maintenance of testing equipment Inadequate service resources	38.14	4	0.207	4
F5	Referral delays	Inadequate healthcare workers Prolonged arrival time of ambulances	34.325	5	0.173	6
F6	Inappropriate rehabilitation services	Inadequate service resources (e.g., equipment, funding and personnel) Impatience of nursing staff	41.641	2*	0.234	2*

≥ average of FRPM (0.2205).

The symbol * indicates The top three items.

The prioritization of Traditional FMEA and Fuzzy FMEA was confirmed by the 13 CMEC experts using an expert questionnaire (Table 11). The difference between the traditional and Fuzzy FMEA-RPN was explained to 13 CMEC experts and then we asked them to mark ‘√' on the two RPN options for finding suitable solutions in accord with solving practical problems. The result represented that 10 CMEC experts agreed with Fuzzy-RPN, which verifies that the applicability of Fuzzy-RPN is better than traditional RPN in solving practical problems.

**Table 11:** Comparison between traditional and Fuzzy FMEA-RPN with the CMEC experts' agreement.

**CMEC experts**	**Traditional RPN**	**Fuzzy-RPN**
Expert 1		√
Expert 2		√
Expert 3		√
Expert 4		√
Expert 5		√
Expert 6	√	
Expert 7	√	
Expert 8		√
Expert 9		√
Expert 10	√	
Expert 11		√
Expert 12		√
Expert 13		√
Percentage of agreement with the experts	**23.07**	**76.93**

Thus, the key service failure points included human resource misallocation, inappropriate rehabilitation services, and slow response by healthcare workers. These three service failure modes were caused by inadequate healthcare workers, prolonged interval between ward rounds, prolonged arrival time of healthcare workers, inadequate service resources, and impatience of nursing staff. To address these service failure situations, we proposed TRIZ inventive principles based on each failure cause in the next phase after analyzing the TRIZ contradiction matrix.

In the third phase, the TRIZ parameters were first mapped and sorted according to the TRIZ correspondence table for CMEC services (see Appendix A) (15, 33). This facilitated the subsequent analysis of the TRIZ contradiction matrix to generate the TRIZ inventive principles. According to Table 9 and Appendix A, the mapping results are as follows.

The most critical service failure was “human resource misallocation,” caused by “inadequate healthcare workers” and “prolonged interval between ward rounds.” These two causes of failure corresponded to service quality determinants No. 13 (number of nursing staff) and No. 5 (service adaptability) in Appendix A, respectively, leading to the corresponding TRIZ improving parameters No. 26 (amount of substance) and No. 35 (adaptability or versatility).

The second most critical service failure was “inappropriate rehabilitation services” caused by “inadequate service resources” and “impatience of nursing staff.” These two causes of failure corresponded to service quality determinants No. 18 (service capacity) and No. 7 (healthcare workers' attitude) in Appendix A, respectively, leading to the corresponding TRIZ improving parameters No. 26 (amount of substance) and No. 17 (temperature).

The third most critical service failure was “slow response of healthcare workers,” caused by “inadequate healthcare workers” and “prolonged arrival time of healthcare workers.” These corresponded to service quality determinants No. 13 (healthcare worker numbers) and No. 20 (time of service delivery time) in Appendix A, respectively, leading to the corresponding TRIZ improving parameters No. 26 (amount of substance) and No. 9 (speed).

In summary, we hoped to improve TRIZ parameters Nos. 9, 17, 26, and 35, but also expected that these improvements would lead to the consumption of additional resources and costs, as well as increase the complexity of the service system. This suggested that the TRIZ worsening parameters No. 22 (loss of energy) and No. 36 (system complexity) should also be considered. In other words, the five failures resulted in “inadequate healthcare workers,” “prolonged interval between ward rounds,” “inadequate service resources,” “impatience of nursing staff,” and “prolonged arrival time of healthcare workers,” which were directly related to greater costs or loss of resources and the increased complexity of system services. Based on the improving parameters Nos. 9, 17, 26, and 35, and worsening parameters Nos. 22 and 36, we derived the TRIZ contradiction matrix (Table 12).

**Table 12:** TRIZ contradiction matrix analysis.

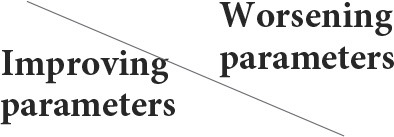	**22 Loss of energy (costs)**	**36 System complexity**
9 Speed	20, 14, 19, 35	10, 28, 4, 34
17 Temperature	21, 17, 35, 38	2, 17, 16
26 Amount of substance	25, 7, 8	3, 13, 10, 27
35 Adaptability or versatility	18, 15, 1	15, 29, 28, 37

Following the TRIZ contradiction matrix analysis, the experts were asked to filter the inventive principles according to their relevance to the attributes of CMEC services. After screening by the experts, seven inventive principles were selected from Table 12 with a high degree of relevance to the attributes of CMEC services, thereby generating inventive solutions: TRIZ inventive principles Nos. 1, 4, 21, 25, 27, 10, and 28.

**Figure 4:**
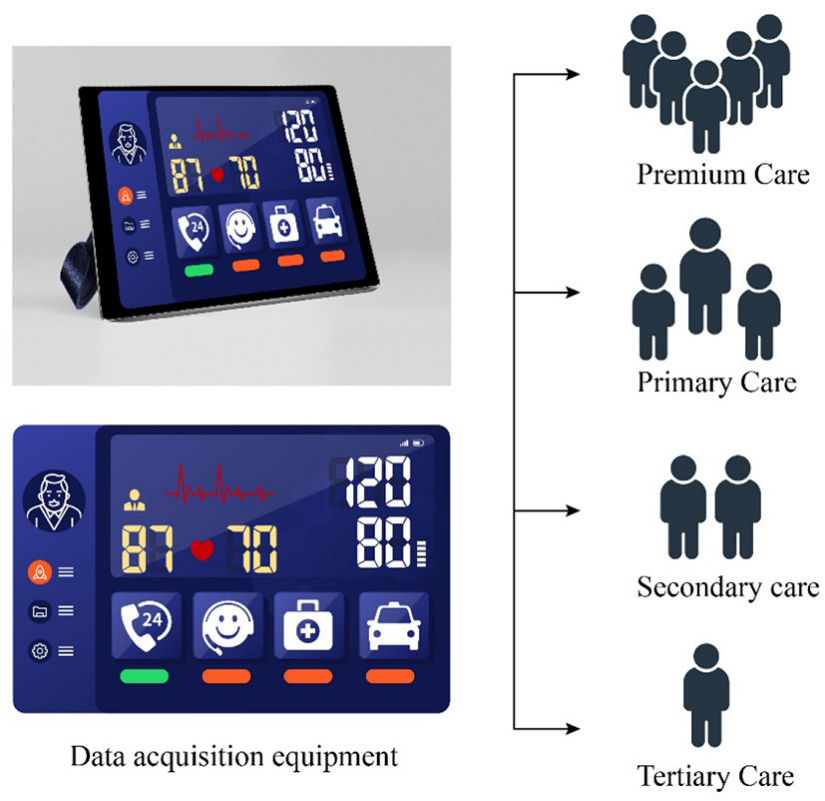
Differential classification of CMEC services.

The TRIZ inventive principle No. 4 (Asymmetry) suggests that the conditions must be changed to increase the asymmetry of an object or system. Owing to the disparities in their health status, elderly people in care facilities have different levels of care needs, possibly affecting the response of healthcare workers. Accordingly, the inventive solution **S1 “Differential distribution of resident healthcare workers according to the level of care needed by the elderly”** was introduced. In the initial stage, physiological data is collected from the elderly upon admission to CMEC institutions, and used for the tiered placement of the elderly in CMEC services (including premium care, primary care, secondary care, and tertiary care), which can then be used as a basis for the differential distribution of resident healthcare workers. After admission, the initial data is combined with the acquisition of real-time physiological data, and the level of care needed by the elderly is analyzed through big data monitoring (Figure 4). This would enable the dynamic classification of care required by the elderly, with fewer healthcare workers stationed near the rooms of elderly with higher self-care capacity, and more healthcare workers placed near the rooms of those who need a higher level of care, thereby decreasing healthcare worker response and arrival times. By increasing the asymmetry in elderly care services and implementing the differential distribution of resident healthcare workers, this principle seeks to enhance the dynamic balance of manpower and resources between “medical care” and “elderly care” services in CMEC workforce.

**Figure 5:**
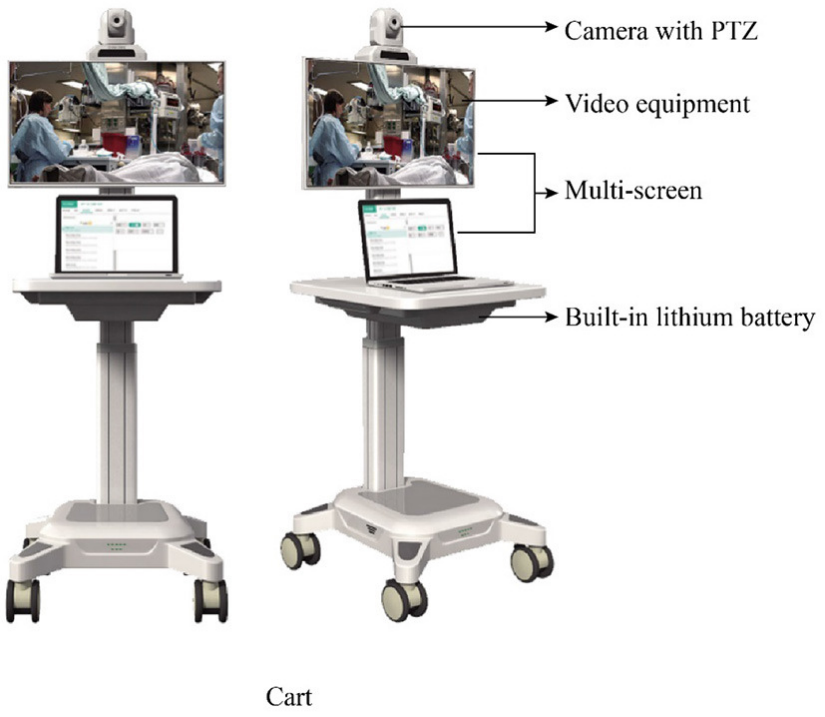
Schematic diagram of CMEC remote ward rounds.

The TRIZ inventive principle No. 21 (Rushing Through) refers to taking steps that are hazardous to the object or harmful to the system at a very high speed. Owing to the lack of staff in CMEC institutions, nursing staff often have to perform other tasks such as cleaning the premises in addition to their daily nursing duties, which affects the speed of nursing response to a certain extent. Accordingly, the innovative solution **S2 “Reduce unnecessary workflows for nursing staff and speed up service completion by establishing remote ward rounds”** was introduced. On the one hand, reducing the workflows of nursing staff beyond the scope of nursing services would reduce service delivery time and enable rapid service completion without affecting the rehabilitation services provided. Furthermore, avoiding response failures by nursing staff would also affect the elderly's perception of service. On the other hand, remote ward rounds can be established (Figure 5) by designing an integrated set of equipment, including a cart, multiple screens, a camera with PTZ, video software, and built-in lithium battery. Remote ward rounds can overcome the geographical constraints involved when healthcare workers are required to physically travel to the elderly's wards. The equipment will support the movement of healthcare workers between different environments, provide real-time access to medical records and vital signs, and offer multi-scenario applications, thereby reducing the waiting time and improving the quality of CMEC services.

**Figure 6:**
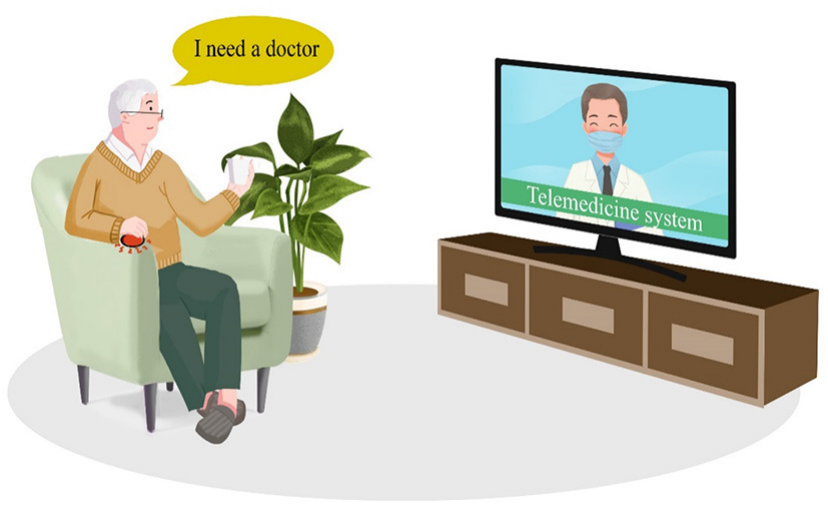
Simplified delivery of CMEC services.

The TRIZ inventive principle No. 25 (Self-Service) suggests that an object or system needs to serve itself in some way. Accordingly, the inventive solution **S3 “Guiding the elderly to voluntarily engage in self-help services”** was introduced. Owing to staff shortages, the promotion of self-help services is essential for CMEC institutions. For elderly people who are fully mobile, healthcare workers can motivate their willingness to engage in self-service through routine instruction and encouragement (Figure 6). For example, in daily life, meals can be served in the cafeteria rather than be delivered to the ward; the delivery of CMEC services can be simplified through a remote system to reduce the difficulty of self-service; or, elderly people who are unwell but fully ambulatory can be encouraged to proactively engage in rehabilitation training and medical check-ups and to pick up medication from the pharmacy independently. Promoting self-service among the elderly would greatly reduce the workload of care services, thus alleviating the problem of shortages in healthcare workers.

**Figure 7:**
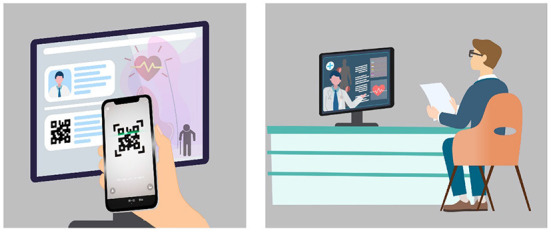
Remote training and teaching for CMEC services.

The TRIZ inventive principle No. 10 (Preliminary Action) suggests performing the required action on an object or system in advance. In other words, elderly care institutions must provide healthcare workers with training required for CMEC services in advance through internships and other means. Accordingly, the inventive solution **S4 “Provide educational training and internships on CMEC services to pre-service healthcare workers”** was introduced. As CMEC services need to combine the functions of “medical care” and “elderly care,” nursing staff are required to acquire more expertise on CMEC, thus placing new demands on pre-service healthcare workers. CMEC institutions could invite pre-service healthcare workers to visit the institution, or launch live training and distance learning sessions at any time through audio and video terminals, which could be viewed by pre-service healthcare workers by scanning the QR code. Pre-service healthcare workers who were unable to attend these sessions could access on-demand learning in the cloud through audio and video terminals, thus improving their experience (Figure 7). In addition, when front-line staff of elderly care institutions would provide care to the elderly, interns could be assigned to participate in this task to acquire more practical experience of CMEC services and the operation of complex medical systems (e.g., telemedicine). This would also help to train staff at all levels to alleviate the shortages in healthcare workers.

**Figure 8:**
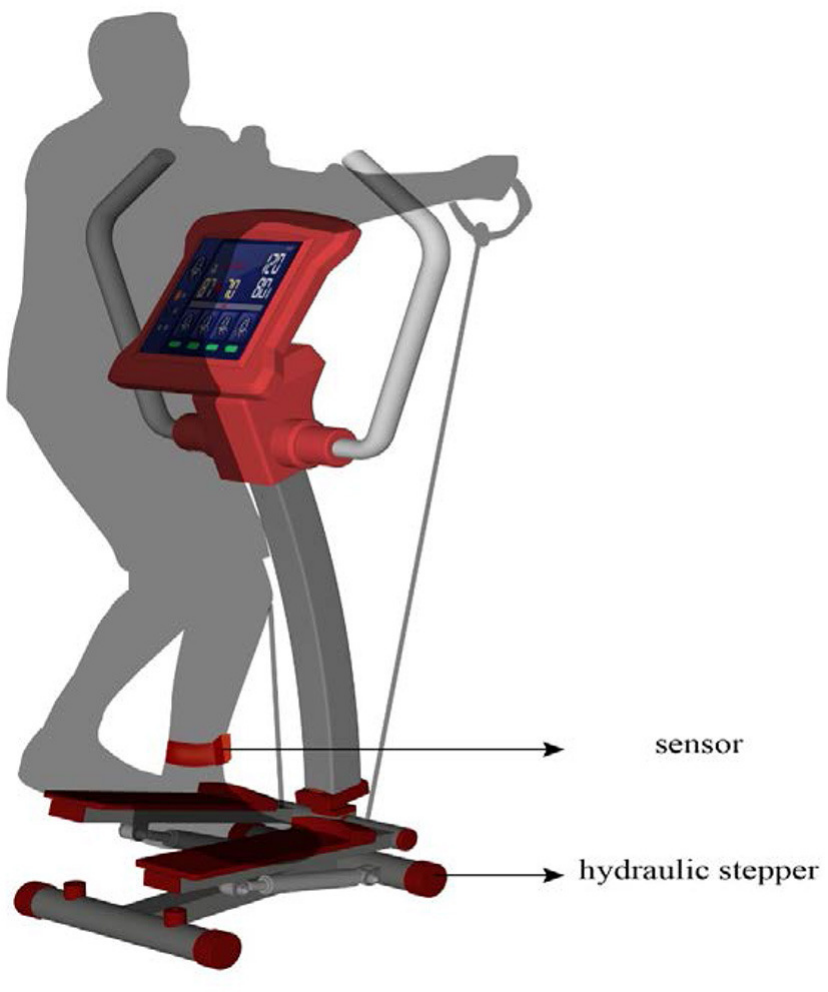
Hydraulic stepper for CMEC services.

The TRIZ inventive principle No. 27 (Cheap Short-Living Objects) implies replacing more resource-intensive parts and steps of an object or system with less resource-intensive parts or steps. Accordingly, the inventive solution **S5 “Use of inexpensive devices and methods for CMEC services”** was introduced. The rehabilitation services in CMEC should be provided using professional training devices. However, inexpensive rather than expensive devices could be used without affecting their established functions, while improving these devices to suit the requirements of CMEC services. For example, a rehabilitation electric bicycle (market price of over RMB 100,000 per unit) can be replaced with a hydraulic stepper (market price of around RMB 5,000 per unit), as both can be used for lower limb training. The hydraulic stepper can also be equipped with sensors for real-time monitoring (Figure 8) to prevent falls and collect exercise data, which will reduce the need for healthcare workers to accompany the elderly during rehabilitation activities. In addition, the institution can provide customized rehabilitation training programs to enhance the lower limb muscle strength and cardiopulmonary function of the elderly by combining their symptoms with the acquired rehabilitation data. This solution reduces capital and labor costs, while meeting the need for lower limb training services, thereby enhancing the institution's elderly care capacity as part of its CMEC services.

The TRIZ inventive principle No. 1 (Segmentation) implies dividing an object or system into several parts to optimize the whole. Accordingly, the inventive solution **S6 “Segmentation of some services in CMEC institutions for outsourcing”** was introduced. General elderly care institutions must consider the needs of the elderly while completing convalescence services. Hence, they are unable to fully meet the needs of the elderly, which can lead to problems such as having prolonged intervals between ward rounds.

Therefore, CMEC institutions can outsource some of their services to service providers located in their vicinity, including non-essential services such as catering (the institution is only responsible for delivery), physical examinations (blood tests, urine tests, etc.) and dialysis treatment. For example, outsourcing catering can strengthen the professionalism of the institution's CMEC services, and outsourcing medical examination services and dialysis treatment to hospitals near the institution can reduce its capital investment by at least RMB 20 million. This solution allows the institution to meet the needs of the elderly for CMEC, while minimizing the service time and operating costs of all aspects of CMEC services, reducing the service content provided by healthcare workers and capital investment, and shifting the focus toward handling the medical care and rehabilitation services of the elderly.

**Figure 9:**
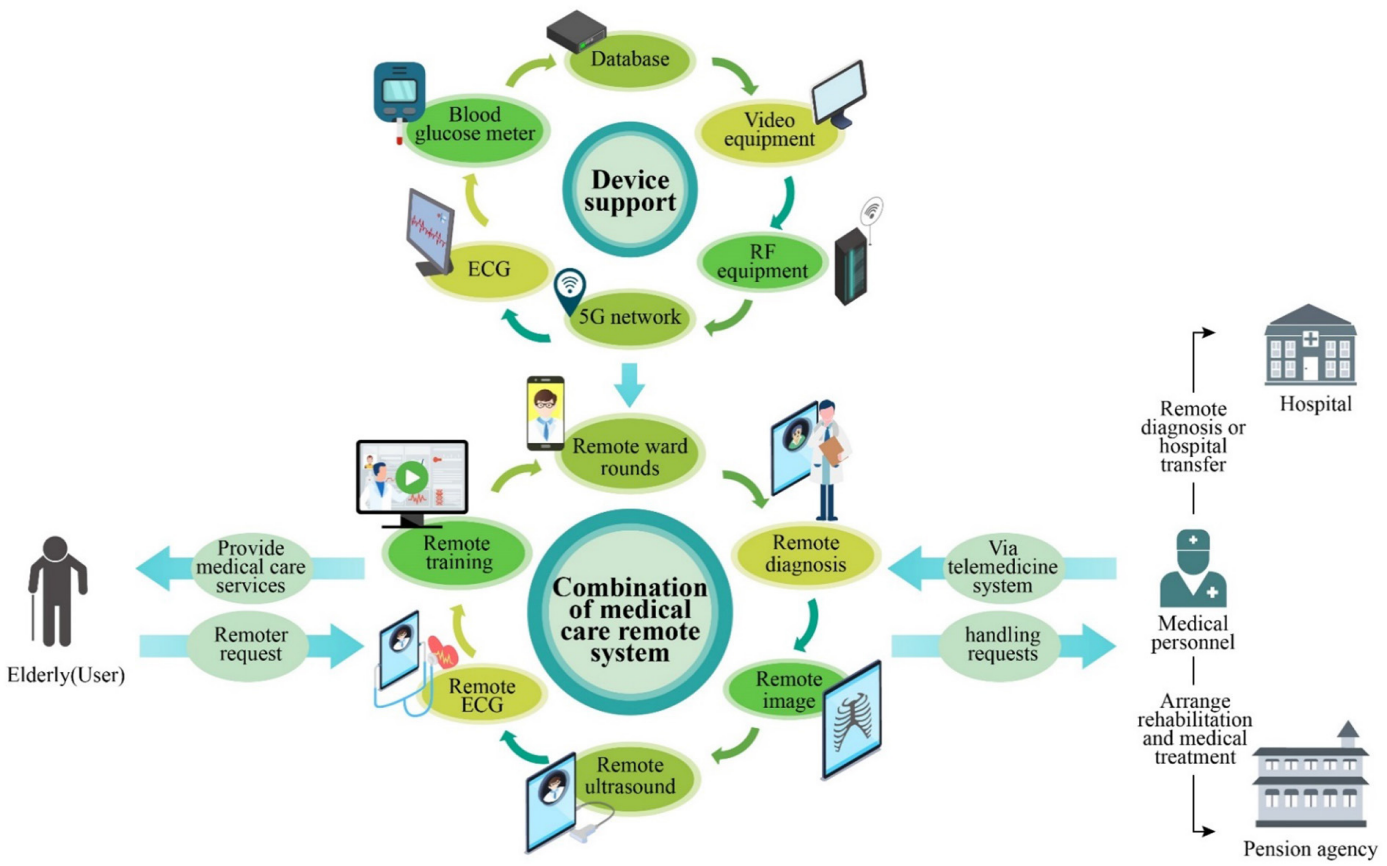
CMEC telemedicine system.

The TRIZ inventive principle No. 28 (Replacement of Mechanical System) refers to the replacement of simple and traditional mechanical fields and mechanical systems with other fields and systems. Accordingly, the inventive solution **S7 “Building intelligent wards with telemedicine systems” was introduced**. By integrating existing medical equipment (e.g., blood glucose meter, electrocardiogram, and rehabilitation equipment) in the ward through a wireless network, surveillance cameras, radio frequency identification equipment, smart wearable equipment, and so on, a CMEC telemedicine system (including teleconsultation, teleradiology, tele-ultrasound, remote ward rounds, and tele-electrocardiogram) can be constructed (Figure 9) to provide intelligent services and improve service efficiency. Real-time testing can be performed on the elderly through a series of body monitoring devices, and the timely detection and diagnosis of diseases can be achieved through big data. With networked and intelligent ward rounds, an elderly person who is suffering from a condition but cannot travel to the hospital can receive remote medical care, thus resolving the spatial constraints of referrals and the limitations in medical resources, while reducing the possibility of service failures leading to the aggravation of the elderly person's condition.

Finally, Table 13 summarizes the seven new service optimization solutions for CMEC services inspired by the TRIZ inventive principles.

**Table 13:** Translation of TRIZ principles into service optimization solutions.

**Inventive principles**	**Service optimization solutions**
No. 4: Asymmetry	S1. Differential distribution of resident healthcare workers according to the level of care needed by the elderly.
No. 21: Rushing Through	S2. Reduce unnecessary workflows for nursing staff and speed up service completion by establishing remote ward rounds.
No. 25: Self-Service	S3. Guiding the elderly to voluntarily engage in self-help services
No. 10: Preliminary Action	S4. Provide educational training and internships on CMEC services for pre-service healthcare workers
No. 27: Cheap Short-Living Objects	S5. Use of inexpensive devices and methods for CMEC services
No. 1: Segmentation	S6. Segmentation of some services in CMEC institutions for outsourcing
No. 28: Replacement of Mechanical Systems	S7. Building intelligent wards with telemedicine systems

## Policy implications

Additionally, the policy recommendations based on the research finding were developed in this study for the Jiangsu provincial government, such as the perspectives of multiple stakeholders (institutional interests, frontline healthcare workers, families of the elderly, and the government) were considered when reviewing service failures and developing solutions to improve service processes. These experiences and opinions can be viewed as key factors in determining the quality of services, optimizing service processes, and ensuring the effectiveness of improvement models. We recommend that the government should take the lead in overall regional planning, position various services offered by CMEC institutions, integrate service resources through public-private partnerships, and conduct the joint management of resources under a government framework. The government's promotion of collective elderly care may help reduce the average cost of elderly care, thereby improving the accessibility and standards of CMEC services and alleviating the lack of resources for the elderly within the context of an aging population.

Furthermore, the managerial participants involved in the case study of elderly care institutions have expressed that this model can facilitate the optimization and improvement of their service system through a set of scientific and systematic methods. In particular, the service blueprint provides an effective analytical approach to help review the entire service process. Unlike production processes in manufacturing, the service blueprint emphasizes a service-oriented approach to assist managers in redesigning and optimizing their service processes. In addition, Fuzzy-FMEA can help managers develop solutions more precisely while eliminating ambiguity and incorporate expert assessment of the proposed solutions. Therefore, managers should establish a management mechanism to inspire diverse optimization solutions, which would enable institutions to continuously optimize their service processes within the context of CMEC for the elderly.

Finally, the service optimization solutions were generated, and we invited the managers and front-line staff of elderly care institutions to evaluate the value of these solutions. The optimization solution **S2**, generated by the inventive principle “Rushing Through,” proposes the speedy completion of services by establishing remote ward rounds, which can reduce the burden of ward rounds for nursing staff and shorten the intervals between ward rounds. This optimization solution allows the institution to decrease the ward round interval from 6 h/ward round to 1 h/ward round or less and reduces the nursing staff's response time by approximately 30%. The optimization solution **S3**, generated by the inventive principle “Self-Service,” proposed that the willingness of the elderly to engage in self-service should be motivated through routine instructions and encouragement. This optimization solution is expected to reduce the routine care work of nursing staff by 25%, which would significantly alleviate the work pressure on nursing staff and the shortage of human resources in the institution, thus allowing more resources to be invested in CMEC services. The optimization solution **S7**, generated by the inventive principle of “Replacement of Mechanical Systems,” requires a certain amount of capital investment and integrated research and development in the initial stages. However, as a long-term investment, it can transform manual healthcare into 24/7 intelligent monitoring, and promote more comprehensive CMEC services when combined with telemedicine systems. Based on the assessment results of institutions that have adopted such systems, this optimization solution can reduce the demands on healthcare workers by 20–30% and increase client satisfaction by 20%.

## Conclusions

The integrated service blueprint-TRIZ model proposed in this study is a systematic method of service optimization design that integrates service blueprint, Fuzzy-FMEA, and TRIZ to realize the connections among the service environment, problem solving, and technological application. Based on this model, we effectively proposed a systematic integration solution for the process optimization of CMEC services, thus providing a micro-level perspective and theoretical guidance for the development of CMEC services in China and effectively alleviating the imbalance of resources between medical care and elderly care services in China's aging society.

Our service design methodology was refined to achieve the feasibility of applying service blueprint and TRIZ based theories in the field of care services. Compared to other service design methodologies (service blueprint, FMEA, QFD, etc.), the integrated service blueprint-TRIZ model is more scientific and advanced with respect to defining the service problems as well as generating and evaluating solutions. Furthermore, it employs fuzzy functions to eliminate the crisp boundaries between the five evaluation levels of the five-point Likert scale. Instead, it divides the evaluation levels of managers into the evaluation of adjacent levels, while using the degree of membership of the triangular fuzzy function to reflect the degree to which the evaluation levels belong to the evaluation of adjacent levels, thereby addressing the uncertainty arising from the evaluation of service failure modes by the managerial participants. The advantages of the integrated service blueprint-TRIZ model are summarized as follows.

(1) The integrated service blueprint-TRIZ model combines the service blueprint with Fuzzy-FMEA to resolve the ambiguity in the evaluation of service failure modes by managers, thus enabling service designers to clearly understand potential service failures and analyze the causes and effects of each service failure mode in the service process.
(2) Instead of relying on the intuition and personal experiences of the service designer, the integrated service blueprint-TRIZ model uses the TRIZ contradiction matrix to eliminate conflicts and generate appropriate inventive principles for the service conceptualization process.
(3) The integrated service blueprint-TRIZ model offers expert-centered screening of inventive principles, service solutions based on the selected inventive principles, and ideal optimization solutions.

Specifically, the academic contributions of this study are as follows: (1) investigation of the Fuzzy-FMEA method for the quantification and measurement of service failures; (2) investigation of service system optimization design, especially the systematic research on the optimization design of CMEC services for the elderly; (3) design of service system optimization strategies based on the actual needs of elderly care institutions for CMEC in Huai'an City, Jiangsu Province; and (4) expansion of service innovation research in the field of CMEC services for the elderly based on previous literature.

The integrated service blueprint-TRIZ model with fuzzy-FMEA approach that was proposed and validated to answer the research questions and bridge the research gaps mentioned in the introduction section. As such, (1) qualitative and quantitative methods were incorporated in fuzzy-FMEA and TRIZ for developing new service conceptualization in initiated from a service failures diagnosis; (2) an efficient elicitation of the new service optimization solution could be achieved by TRIZ; and (3) the abundant fuzzy elements, including the triangular fuzzy number, fuzzy linguistic variables, and fuzzy probability range were utilized in the proposed model for eliminating arbitrariness, ambiguity, and uncertainty from expert judgment. Meanwhile, a comparison of the integrated service blueprint-TRIZ model with the existing studies is described clearly in Table 14. A better understanding of the research novelty could be achieved.

**Table 14:** The novelty of the present study used in CMEC compared with the existing studies.

**Source**	**Topic**	**The previous studies**
Yang et al. (2); Wei and Zhang (4); Penkunas et al. (14); Zolfaghari and Mousavi (21); Chowdhury and Quaddus (34); Behdioglu et al. (35); Sun et al. (36); Karami et al. (37); Xu et al. (38)	Hospital service quality survey	* The fuzzy method was used largely in evaluating hospital service quality and elderly's requirement. * The triangular fuzzy number was utilized to eliminate an ambiguous expression of the decision-makers' opinions for service quality improvement. * A fuzzy probability distribution was integrated into the decision-making attributes of the expert. * The fuzzy linguistic variables were utilized for measuring service quality from the attitude of decision makers. * The service process and quality evaluation in hospital was conducted, the method includes: SERVQUAL, Kano model, AHP, ANP, TOPSIS, and QFD. * The triangular fuzzy number was applied (e.g., QFD and AHP) to calculate the relationship between customer requirement and service design strategies.
**Source**	**Topic**	**The novelty of the present study**
Present study	CMEC service optimization and in*nova*tion	* The integrated service blueprint-TRIZ model was proposed in this study for optimizing the combined medical and elderly care services (CMEC). * The fuzzy-FMEA incorporating qualitative and quantitative methods was used in CMEC service failure diagnosis. * The CMEC service blueprint was established and its potential service failures were recognized. * The ambiguous expression of the CMEC managers was eliminated by the fuzzy-FMEA approach. * A systematic innovation method for CMEC service was proposed to facilitate service designers generating suitable innovative principles for the new service optimization solutions. * The new service optimization solutions were realized for optimizing CMEC service efficiency.

Finally, this study used the TRIZ inventive principles to inspire seven inventive solutions for improving service processes. These inventive solutions can be applied in practice within the field of CMEC services. As this was an exploratory study, its scope is limited to the systematic design of a decision-making model and field of elderly care services. Therefore, further investigations in other related areas should be conducted in the future.

## Data availability statement

The original contributions presented in the study are included in the article/Supplementary material, further inquiries can be directed to the corresponding author.

## Ethics statement

Ethical review and approval was not required for the study on human participants in accordance with the local legislation and institutional requirements. Written informed consent from the participants was not required to participate in this study in accordance with the national legislation and the institutional requirements.

## Author contributions

A-JS: writing—original draft and revising the manuscript, research conceptualization, methodology, supervision, and data analysis. W-FW: investigation, data analysis, coordinating tasks, and writing—revising the manuscript. MY: formal analysis and validation, investigation, writing—revising the manuscript, and final approval of the version. XW and HL: research administration for the empirical project, resources, interpretation of data, writing—revising the manuscript, and final approval of the version. All authors read and approved the final manuscript and agreed to be accountable for all aspects of the work in ensuring that questions related to the accuracy or integrity of any part of the work are appropriately investigated and resolved.

## Funding

This research was supported by the Social Science Fund of Jiangsu Province [Grant Number: 19SHB003], Natural Science Foundation Fund of Fujian Province [Grant Number: 2022J01310], and the starting research fund for higher-level talents from Huaqiao University [Grant Number: 21SKBS007].

## Conflict of interest

The authors declare that the research was conducted in the absence of any commercial or financial relationships that could be construed as a potential conflict of interest.

## Publisher's note

All claims expressed in this article are solely those of the authors and do not necessarily represent those of their affiliated organizations, or those of the publisher, the editors and the reviewers. Any product that may be evaluated in this article, or claim that may be made by its manufacturer, is not guaranteed or endorsed by the publisher.
